# Microbiological profile and prevalence of histamine-producing bacteria in fresh sardines stored at different temperatures

**DOI:** 10.14202/vetworld.2024.2376-2384

**Published:** 2024-10-31

**Authors:** Rachid Khatouf, Said Dahani, Oleya El Hariri, Rajaa Amiyare, Nourredine Bouchriti

**Affiliations:** 1 Department of Veterinary Pathology and Public Health – Food safety unit Hassan II Agronomic and Veterinary Institute, Rabat, Morocco; 2 Laboratory of Biochemistry, Biotechnology, Health and Environment, Department of Biology, Faculty of Science, University Ibn Tofail, Kenitra 14999, Morocco

**Keywords:** *Enterobacteriaceae*, histamine, Histaminogenic bacteria, polymerase chain reaction, sardine, seafood

## Abstract

**Background and Aim:**

The European pilchard (*Sardina pilchardus*) is an important fish species for the Moroccan economy in terms of production and export. Biogenic amine histamine is a metabolite produced in the flesh of some fish species after death due to the decarboxylation of free histidine by histaminogenic bacteria. Failure to control the histamine risk in European pilchard may lead to public health and socioeconomic issues. This study aimed to estimate the prevalence of histaminogenic bacteria in association with histamine levels and the growth of microflora in Moroccan sardines (European pilchard).

**Materials and Methods:**

We conducted the study by monitoring Moroccan sardines of histamine content and microbiological profile (aerobic plate count [APC], coliforms, and thermo-tolerant coliforms [TTC]) during 6 days of storage at three different temperatures (0°C, 10°C, and ambient temperature [22°C]). The histamine assay was performed using a spectrofluorometric method, and the microbiological identification of histamine-producing bacteria was performed using a combination of biochemical and molecular tests.

**Results:**

The histamine content in European pilchard stored at 0°C was negligible. However, high concentrations were observed at 10°C and 22°C. The microbiological profile showed a positive association between microflora counts and histamine content according to storage time. At 0°C, a moderate increase in the APC, a decrease in coliforms, and an absence of TTC were observed. The rapid proliferation of all microflora was observed at 10°C, whereas at 22°C, the proliferation was almost exponential. Bacterial identification revealed the exclusive presence of species belonging to the *Enterobacteriaceae* family at varying frequencies depending on storage temperature. *Morganella morganii* and *Proteus mirabilis* had the highest histamine induction rates in L-histidine-supplemented broth, with 1600 and 255 parts per million (ppm), respectively, after 48-h incubation at 35°C. *Klebsiella ozaenae* could produce 136 ppm and *Serratia plymuthica* 115 ppm. Reverse transcription polymerase chain reaction showed positive results for the presence of genes associated with histidine decarboxylase. The *hdc* genes of *M. morganii*, *P. mirabilis*, and *K. ozaenae* were successfully amplified and exhibited strong similarity with the reference gene of *M. morganii*.

**Conclusions:**

This study describes for the first time the *hdc* gene in bacteria that form histamine in Moroccan sardines. The results also confirm that respect for the cold chain integrity is a crucial factor in histamine management. This information should help stakeholders in the implementation of sound strategies for managing the hazards associated with seafood and their products.

## Introduction

The European pilchard (*Sardina pilchardus* Walbaum, 1792) is a fish rich in protein but very perishable [1, 2]. It is considered a fish species rich in free histidine [3, 4]. This free amino acid is transformed into histamine by histidine decarboxylase (HDC), an enzyme excreted by bacteria during the breakage of the cold chain [5]. The excretion of this enzyme automatically triggers the induction of histamine even if the bacteria involved are eliminated. Once produced, no additional treatment can get rid of it [5]. Excessive amounts of histamine cause scombroidic poisoning [6]. Histamine is the most toxic biogenic amine; its neurotransmitter and vasodilator action induces headaches, redness, and cutaneous edema in the face and neck, a drop in blood pressure and cardiac disorders, asthma attacks, and respiratory distress in addition to damage to the gastrointestinal system (nausea and diarrhea) [3, 7, 8]. Between 2008 and 2010, 51% of foodborne illnesses linked to fish products in Europe and 20% in the US were due to the consumption of fish containing high levels of histamine (>500 mg/kg) [8]. Many countries have established maximum levels of histamine that should not be exceeded in food intended for human consumption because of its harmful effects. It is one of the most studied biogenic amines for which a specific regulation has been implemented worldwide [7]. The histamine control requires knowledge of the bacterial strains involved in its formation in sardines and the study of their presence during storage at different temperatures. Most of the histamine-inducing bacteria in fish are enterobacteria including *Morganella morganii, Raoultella ornithinolytica, Raoultella planticola, Proteus vulgaris, Proteus mirabilis, Klebsiella* spp *., Enterobacter cloacae, Enterobacter aerogenes, Citrobacter freundii, Serratia liquefaciens*, and *Serratia fonticola* [8–11].

Identification of histamine-forming bacteria is the fundamental objective of scombroid poisoning research. Niven medium was introduced to isolate the bacteria. However, many false positive and false negative results were obtained using this medium even after certain modifications [8]. Selective media have also been tested, but their effectiveness in detecting potential histamine trainers remains questionable [12]. Subsequently, molecular biology methods were implemented to identify histamine-producing bacteria in fish by amplifying the gene regulating the secretion of HDC and partial or whole sequencing of rDNA [8, 13].

The safety and quality of fresh fish are impacted by handling conditions and storage temperature, which can differ, as they can cause the growth of bacteria. To our knowledge, few studies have investigated the molecular characterization of histamine-producing bacteria in Moroccan sardines [14, 15].

Therefore, this study aimed to estimate the prevalence of histaminogenic bacteria in association with histamine levels and the growth of microflora in Moroccan sardines (European pilchard) at three different temperatures (0°C, 10°C, and at ambient temperature [22°C]). Biochemical and molecular methods were used to identify bacteria implicated in histamine production. The results of this study will provide essential information for fishing industry stakeholders and concerned authorities to implement targeted and effective control measures to reduce bacterial contamination and prevent food poisoning related to sardine consumption. In addition, this study will generate new knowledge about histaminogenic bacteria and the organization of genes encoding histamine induction in fish.

## Materials and Methods

### Ethical approval

This research did not involve animal or human participants, nor did it collect personal data. So, ethical approval was not necessary.

### Study period and location

The study was conducted from February to June 2023 in the fish safety laboratory of the Food Safety Unit, Department of Veterinary Pathology and Public Health of the Hassan II Agronomic and Veterinary Institute, Rabat, Morocco.

### Samples

Three randomly selected batches of sardines from three different boats were collected at a landing point at the Port of Mehdia located on the North Atlantic coast of Morocco (Food and Agriculture Organization fishing area 34). The samples were packaged under ice in Styrofoam boxes and sent directly to the laboratory, where each batch was divided into three groups (A, B, and C); Group A was stored under ice in a refrigerator at 0°C, Group B was stored in a refrigerator at 10°C, and Group C was left at 22°C. Groups A and B were kept for 6 days, and Group C was kept for 2 days. A total of 120 samples were used, 51 each for Groups A and B and 18 for Group C.

### Temperature measurement

The core temperature of each group of fish was checked every 8 h using a calibrated probe thermometer (Testo 110, Forbach, France). The arithmetic mean of the core temperature for each batch was calculated.

### Histamine content

The histamine assay was carried out on samples from each group and was performed every 8 h during storage using a spectrofluorometric method [16]. The first assay was performed approximately 9 h after capture. Histamine was extracted from 10 g of minced flesh, mixed with 90 ml of a solution of trichloroacetic acid, separated by chromatography on an ion-exchange column (Amberlite CG50), and eluted from the column with 0.7 N hydrochloric acid. The assay was performed by fluorometry after complexation with orthophthalaldehyde. Histamine concentrations were measured using a fluorometer Trilogy™ (Turner Designs Instrument, model 7200-000, California, USA) by fluorescence at emission and excitation wavelengths of 450 and 360 nm, respectively.

### Microbial counts and identification of histamine-producing bacteria (HPB)

The evolution of bacterial microflora was monitored in flesh fish from all groups after 2, 4, and 6 days of storage at 0°C and 10°C and after 12, 24, and 36 h of storage at 22°C. The search for aerobic plate count (APC), which aims to determine the quantity of bacteria present in the samples and to detect the effect of possible poor storage conditions, was carried out on plate count agar (Biokar, France) seeded in double layers and incubated at 30°C for 72 h aerobically. Coliforms, which are enterobacteria living as commensals in the intestines of warm-blooded animals and which are indicated by fecal contamination of food, were carried out on Violet Red Bile Lactose medium (VRBL) (Biokar), with double-layer incubation at 30°C for 24 h and thermo-tolerant coliforms (TTC) on VRBL medium, with double-layer incubation at 44°C for 24 h. The glucose-fermenting enterobacteria were isolated on Violet Red Bile Glucose (VRBG) (Biokar) medium, with incubation in a double layer at 30°C and 44°C for 24 h. All inoculations were performed in duplicate with 0.1 ml of the stock suspension and decimal dilutions.

The microflora likely to produce histamine were isolated and identified by subculturing on Tryptone Soya Agar (TSA) (Biokar), as indicated by individualized colonies surrounded by a purple halo in the VRBG countable boxes. Phenotypic identification tests (oxidase test using oxidase disk, gram staining, and morphology) were used for preliminary classification of the isolated strains, followed by the commercial bacterial identification system Analytical Profile Index (API) 20 E Gallery (Biomérieux, France) for bacterial identification.

### Histamine induction power

The ability of isolated strains to induce histamine production was studied in Heart-Brain Infusion (BHI) (Biokar) broth supplemented with 1% free L-histidine. A loop of a fresh bacterial culture for 24 h was taken from the TSA medium of each strain, and it was mixed with 10 mL of BHI infusion, adding 1% L-histidine. Histamine production was determined using the Lerke–Bell fluorimetric method after 48 h of incubation at 35°C. Isolated strains confirmed to induce histamine were identified at the species level using the Phoenix™ instrument (Becton Dickinson, USA), according to the manufacturer’s recommended protocol.

### DNA extraction and real-time reverse transcription polymerase chain reaction (RT-PCR)

The genomic DNA of the isolated bacteria was extracted using an Invitrogen PureLink Genomic DNA Mini Kit (Thermo Fisher Scientific, USA) according to the manufacturer’s instructions. Each sample of extracted DNA was dosed at optical density using a Nanodrop ND-1000 spectrophotometer (Thermo Fisher Scientific). The specific primers of the *hdc* gene described by Takahashi *et al*. [13] and Podeur *et al*. [8], *hdc*-forward (5’-TCH ATY ARY AAC TGY GGT GAC TGG RG-3’) and *hdc*-reverse (5’-CCC ACA KCA TBA RWG GDG TRT GRC C-3’) were used for real-time PCR using an Xpert One-Step Fast SYBR (Applied Biosystems™, Thermo Fisher Scientific) as a screening test to determine the presence of *hdc* genes. The reaction mixture contained 10 μl of Fast qPCR Master mix (SYBR), 15 μL of nuclease-free water, 0.8 μL of each primer to a final concentration of 10 μM, and 5 μL DNA template. The reaction was carried out on a 7500 Real-Time System (Applied Biosystems, Foster City, CA, USA) according to the following protocol: 95°C for 10 min followed by 40 cycles of 95°C for 2 min, 60 for 20 s, and 65°C for 30 s.

### Conventional PCR and DNA purification

Samples positive by real-time PCR were subjected to *hdc* gene amplification using the reaction volume contained in 25 μL of DreamTaq Green PCR Master Mix (Taq polymerase, MgCl_2_, optimized buffer, deoxyribonucleotides triphosphates), 18 μL of nuclease-free water, 1 μL of each primer to a final concentration of 10 μM and 5 μL of DNA extracted. This mixture was introduced into a thermocycler with the following parameters: (preliminary denaturation at 95°C for 5 min, 35 cycles: 95°C for 1 min for denaturation, 55°C for 1 min for hybridization, and 72°C for 1 min for elongation, followed by a final extension step at 72°C for 10 min).

The *16s rRNA* genes were amplified using forward 16s rRNA (5’-ACG-GTG-ACT-AGG-TGT-GGG-TTT-C-3’) and reverse 16s rRNA (5’-TCT-GCG-ATT-ACT-AGC-GAC-TCC-GAC-TTC-A-3’) primers. PCR was performed using the DreamTaq Green PCR Master Mix solution. The PCR reaction was performed in 30 μL reaction mixture containing 25 μL Master Mix (Optimized buffer, Taq polymerase, Deoxyribonucleotides triphosphates, MgCl2), 1 μL of each primer with final concentration 10 μM, 18 μL nuclease-free water, and 5 μL extracted DNA. The mixture was amplified into a thermocycler with the following parameters: Preliminary denaturation at 94°C for 5 min, 25 cycles of 94°C for 1 min for denaturation, 60°C for 1 min for hybridization, and 72°C for 1 min for elongation, followed by a final extension step at 72°C for 10 min.

The amplification products were analyzed on a 1% agarose gel. Subsequently, the amplicons were extracted from the gel and purified using a Nucleospin Gel and PCR Clean-up kit (Macherey-Nagel, Germany). Purified PCR products were used as templates for sequencing using the BigDye^®^ Terminator v1.1 Cycle Sequencing Kit (Life Technologies, Thermo Fisher Scientific). The second purification step was performed using the Big Dye XTerminator Purification kit (Life Technologies). Purified PCR products were sequenced from both directions using the same primers.

### Nucleotide sequences and deduced amino acid sequences

Assembly and analysis of sequence data were performed using BioEdit software version 5.0.9 (https://bioedit.software.informer.com/5.0/) [17]. The open-source BLAST program (National Center for Biotechnology Information, Bethesda MD, http://blast.ncbi.nlm.nih.gov/Blast.cgi) was used for sequence comparison. Nucleotide sequences and deduced amino acid sequences were aligned usingClustalW (European Bioinformatics Institute (EBI), https://www.ebi.ac.uk/Tools/msa/clustalw2/) and MEGA software Version 6.0 (Center for Evolutionary Medicine and Informatics, Arizona State University, https://www.megasoftware.net/) [18]. Phylogenetic analyses and tree construction for partial S1 glycoprotein gene sequences were performed using the neighbor-joining method with 1,000 bootstrap replicates using MEGA.

## Results

### Temperature monitoring

Temperature monitoring of European pilchard stored at 0°C showed variation between 0.1°C and 1°C; the average temperature was 0.49°C. At 10°C, the temperature varied between 8.2°C and 10.7°C with an average of 9.81°C. At 22°C, the results varied between 21°C and 24.2°C with an average of 22.76°C.

### Histamine production during storage

**Figure-1:**
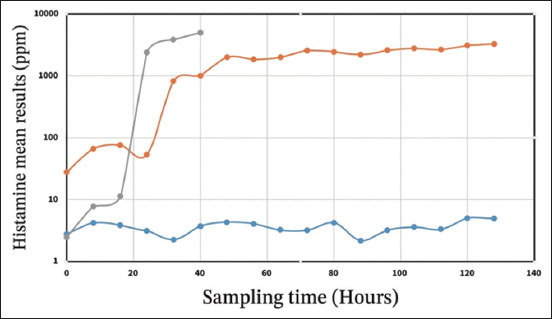
Histamine content (ppm) in European pilchard Stored at 0°C (blue), 10°C (orange), and ambient temperature (22°C) (gray).

Figure-1 illustrates the change in histamine content with storage temperature. Histamine was stabilized between 2 and 5parts per million (ppm) throughout the 6 days in sardine stored at 0°C. At 10°C, histamine levels rapidly evolved to high concentrations of approximately 824 ppm from the 32^nd^ h of storage. Histamine levels increased significantly and exceeded 1900 ppm on the 3^rd^ day of storage. At 22°C, the histamine concentration evolved exponentially and exceeded the regulatory limit (200 ppm) after 24 h of storage.

### Microbial counts during storage at different temperatures

#### APC

**Figure-2:**
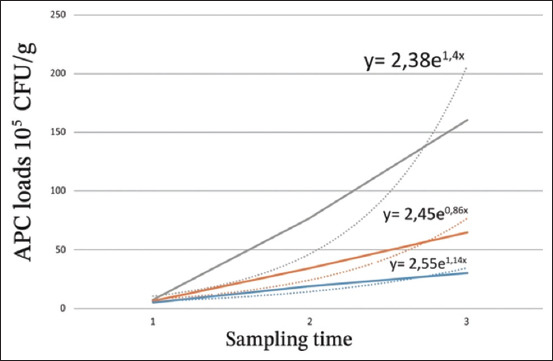
Aerobic plate count loads at different storage temperatures (10^5^ colony-forming unit/g). 1, 2, and 3 correspond, respectively to samples stored for 48, 96, and 144 h at 0°C and 10°C and at 12, 24, and 36 h at ambient temperature (22°C). Blue: Storage at 0°C; orange: Storage at 10°C; gray: Storage at 22°C.

Table-1 summarizes the results of the APC count at different temperatures, and Figure-2 illustrates the evolution of this count, which exhibits an exponential trend, as shown by the exponential regression equation. At 0°C, the average APC increased significantly from 5.3 × 10^5^ colony-forming unit (CFU)/g after 48 h of storage to 3.10^6^ CFU/g after 144 h of storage. A continuous increase in the APC load was observed.

**Table-1:** APC loads at different storage temperatures (10^5^ CFU/g).

Storage temperature	1	2	3
0°C	5.3	19	30
10°C	6.5	34.3	64.3
Ambient temperature (22°C)	8.2	76.7	160

1, 2, and 3 correspond, respectively, to samples stored for 48, 96, and 144 h at 0°C and 10°C and 12, 24, and 36 h at 22°C. APC=Aerobic plate count, CFU=Colony-forming unit

At 10°C, the average APC ranged from 6.5 × 10^5^ CFU/g after 48 h of storage to 6.4 × 10^6^ CFU/g after 144 h of storage. At 22°C, APC counting showed a significant increase. After 12 h of storage, the APC count was 8.2 × 10^5^ CFU/g and increased to 1.6 × 10^7^ CFU/g after 36 h.

#### Coliforms (C)

**Figure-3:**
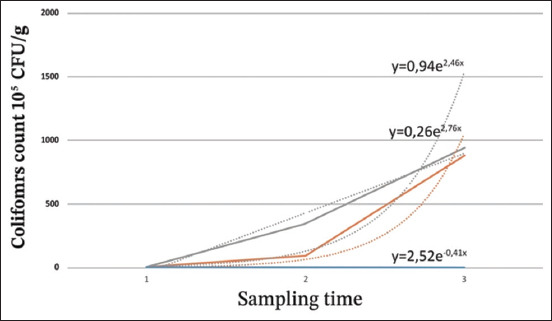
Evolution of coliforms at different temperatures (10^2^ colony-forming unit/g). 1, 2, and 3 correspond, respectively to samples stored for 48, 96, and 144 h at 0 and 10°C and 12, 24, and 36 h at ambient temperature (22°C). Blue: Storage at 0°C; orange: Storage at 10°C; gray: Storage at 22°C.

Table-2 shows the results of the coliform count, and Figure-3 illustrates the evolution. At 0°C, a decrease in coliform loads was observed over time. The means showed a general downward trend during storage, ranging from 1.6 × 10^2^ CFU/g after 48 h to 0.7 × 10^2^ CFU/g after 144 h. At 10°C, coliform count values increased rapidly from 3.5 × 10^2^ CFU/g after 48 h of storage to 8.8 × 10^4^ CFU/g after 144 h. A rapid increase in count values over time was observed at 22°C. Values increased from 6.7 × 10^2^ CFU/g after 12 h of storage to 9.4 × 10^4^ CFU/g after 36 h.

**Table-2:** Coliform count according to storage temperature (10^2^ CFU/g).

Storage temperature	1	2	3
0°C	1.6	1.2	0.7
10°C	3.5	94.3	883
Ambient temperature (22°C)	6.8	347	940

1, 2, and 3 correspond respectively to samples stored for 48, 96, and 144 h at 0°C and 10°C and 12, 24, and 36 h at 22°C. CFU=Colony-forming unit

#### TTC

**Figure-4:**
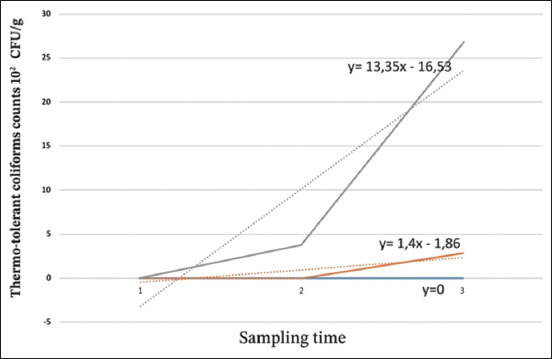
Evolution of thermo-tolerant coliforms at different temperatures and storage times (10^2^ colony-forming unit/g). 1, 2, and 3 correspond, respectively, to samples stored at 48, 96, and 144 h at 0°C and 10°C and at 12, 24, and 36 h of storage at ambient temperature (22°C). Blue: Storage at 0°C; orange: Storage at 10°C; gray: Storage at 22°C.

The results of the TTC count are presented in Table-3, and the evolution of the result is presented in Figure-4. At 0°C, TTCs were absent throughout storage at 0°C and during the first 4 days at 10°C. The loads reached 2.8 × 10^2^ CFU/g after 144 h of storage at 10°C. A rapid increase in count values after 96 h of storage was observed at 22°C. The average increased from 0 CFU/g at 12 h of storage to 3.5 × 10^2^ CFU/g after 24 h of storage and then increased exponentially at 36 h of storage to 2.7 × 10^3^ CFU/g.

**Table-3:** Thermos-tolerant coliform counts at different storage temperatures (10^2^ CFU/g).

Storage temperature	1	2	3
0°C	0	0	0
10°C	0	0	2.8
Ambient temperature (22°C)	0	3.8	26.7

1, 2, and 3 correspond respectively to samples stored for 48, 96, and 144 h at 0°C and 10°C and at 12, 24, and 36 h at 22°C. CFU=Colony-forming unit

### Microbial identification results and histamine induction potency

On countable VRBG Petri dishes, 107 bacterial strains underwent a series of plating by exhaustion to isolate them. Preliminary tests were carried out to target the Enterobacteria family; only negative oxidase, Gram-negative, and bacillary forms were retained. The results of these tests revealed 37 oxidase-positive and 15 oxidase-negative coccoid-shaped strains. Thus, 55 strains were identified as oxidase-negative, Gram-negative, and bacillary, confirming their affiliation to the Enterobacteria family.

The identification of strains using the API20E gallery allowed us to obtain information on the composition of the studied bacterial population. At 0°C, 10 bacteria were identified. Of these, 4 (40%) were identified as belonging to the genus *Enterobacter*, 4 (40%) to *Citrobacter*, 1 (10%) to *Serratia*, and 1 (10%) to *Shigella*. At 10°C, 14 bacteria were identified. Of these, 5 (35.71%) were identified as being of the genus *Enterobacter*, 2 (14.29%) as *Serratia*, 2 (14.29%) as *Shigella*, 2 (14.29%) as *Escherichia* *coli*, 2 (14.29%) as *Proteus*, and 1 (7.13%) was identified as *Citrobacter*. However, at 22°C, 31 bacteria were identified. Of these, 7 (22.58%) bacteria were identified as belonging to the genus *E*. *coli*, 4 (12.90%) as *Enterobacter*, 4 (12.90%) as *Serratia*, 4 (12.90%) as *Citrobacter*, 3 (9.68%) as *Morganella*, 3 (9.68%) as *Providencia*, 2 (6.45%) as *Klebsiella*, 2 (6.45%) as *Proteus*, and 2 (6.45%) as *Shigella*. Table-4 presents the rate frequencies of the presence of isolated strains at 0°C, 10°C, and 22°C.

**Table-4:** Frequency of strains isolated at different storage temperatures.

Strain	0°C	10°C	Ambient temperature (22°C)
*Enterobacter*	4 (40%)	5 (35.71%)	4 (12.90%)
*Citrobacter*	4 (40%)	1 (7.13%)	4 (12.90%)
*Serratia*	1 (10%)	2 (14.29%)	4 (12.90%)
*Shigella*	1 (10%)	2 (14.29%)	2 (6.45%)
*Escherichia coli*	0	2 (14.29%)	7 (22.58%)
*Proteus*	0	2 (14.29%)	2 (6.45%)
*Providentia*	0	0	3 (9.68%)
*Klebsiella*	0	0	2 (6.45%)
*Morganella*	0	0	3 (9.68%)
Total	10	14	31

The genera *Citrobacter, Enterobacter, Shigella*, and *Serratia* were present at all three storage temperatures, with a variable rate of presence. *E. coli* and *Proteus* were present during storage at 10°C and 22°C, while *Morganella* and *Klebsiella* were present only at 22°C.

### Histamine induction power

The histamine induction rate by all isolated strains was determined by fluorometry after histidine infusion. Differences were observed in the ability of histamine induction by these bacteria; *Morganella* had the highest induction rate, with 1600 ppm produced after 48 h incubation at 35°C. *Proteus* could produce 255 ppm, *Klebsiella* 136 ppm, and *Serratia* 115 ppm. The other bacterial strains did not show an important histamine induction power. Indeed, *Providencia* produced 52, *Citrobacter* 35, *Enterobacter* 26, and *E. coli* 16 ppm. Species identification of bacteria with histaminogenic potency was performed using Phoenix™ (Becton Dickinson) and confirmed the identity of the isolated strains. The species identified were: *Providencia rustigianii, M. morganii, Klebsiella ozaenae*, *Serratia plymuthica*, *C. freundii*, and *P. mirabilis*.

### Molecular analysis

#### Extraction and analysis of DNA

DNA was successfully extracted from the strains using the Invitrogen PureLink Genomic DNA Mini Kit. The purity of the extracts was evaluated and revealed a ratio of 260/280; the measured DNA concentrations were higher than 40 ng/μL, attesting to the quality of the extracts.

### Real-time PCR

**Figure-5:**
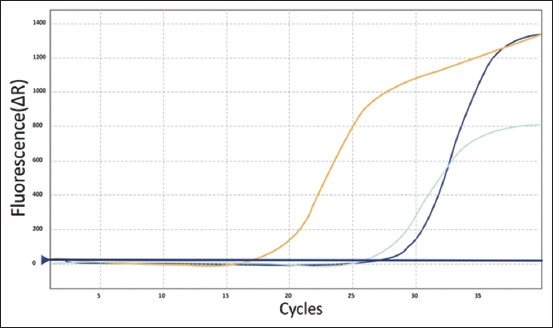
Real-time polymerase chain reaction results.

Real-time RT-PCR was negative for all strains except for *P. mirabilis, M. morganii*, and *K. ozaenae*, of which PCR was positive with a Ct value greater than 30, confirming the presence of genes bound to the HDC enzyme, as shown in Figure-5.

### Conventional PCR

**Figure-6:**
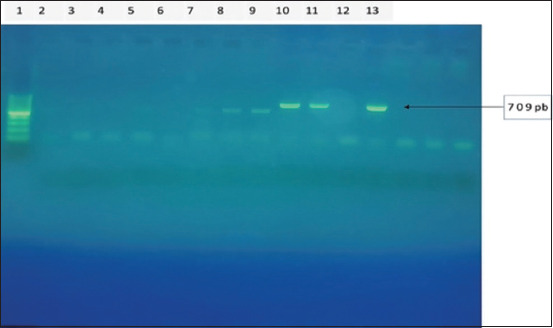
Separation of amplified products by electrophoresis on 1.5% agarose gel (709 pb). 1: Genetic marker of 100 pb. Lanes 2–13 show the polymerase chain reaction-amplified *hdc* gene of respective strains. Lanes 10, 11, and 13 correspond, respectively, to the *hdc genes* of *Morganella morganii, Proteus mirabilis*, and *Klebsiella ozaenae* isolated.

All isolated strains underwent conventional PCR to amplify the *hdc* genes; only the *hdc* genes of the *M*. *Morganii, P. mirabilis*, and *K. ozaenae* strains were successfully amplified. The size of the amplification products during agarose gel migration was consistent with what was expected (about 709 base pairs). As shown in Figure-6, the amplification products were purified and sequenced. The other strains did not generate an amplification product.

**Figure-7:**
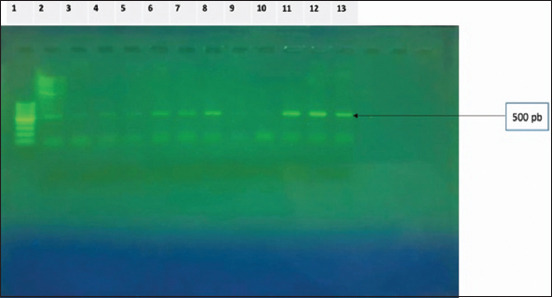
Separation of 16s-amplified products by electrophoresis on a 1.5% agarose gel (500 bp). 1: Genetic marker of 100 pb. Lanes 2–13 show the polymerase chain reaction-amplified *16s* *rRNA* gene of the respective strains isolated.

The phylogenetic analysis was performed using universal primers specific for *16S rRNA* genes. The size of the amplification products matched that expected approximately 500 base pairs, as shown in Figure-7. The amplification products were then purified and sequenced.

### Sequence analysis and alignment

The sequences of the *hdc* and *16S rRNA* genes obtained were analyzed and compared with those deposited in GenBank to explore the degree of similarities. The *hdc* genes of the strains isolated in our study had a very high degree of similarity: 98.67% with *M. morganii*, 98.52% with *P. mirabilis*, and 98.28% with *K. ozaenae*. Regarding the comparison of 16s rRNA sequences, alignment confirmed biochemical identification and membership to the *Enterobacteriaceae* family with significant degrees of similarity (93.8%–96.98%), as shown in Table-5.

**Table-5:** Percentage similarity between isolated strains and GenBank.

Species in GenBank	Similarity (%)	Accession number
*E. hormaechei* subsp.*xiangfangensis*	94.95	MG461544.1
*E. cloacae*	94.03	KT261190.1
*S. plymutica*	94.12	KJ729609.1
*P. vulgaris*	94.90	KF657723.1
*M. morganii*	95.57	KY606572.1
*K. pneumoniae* subsp.*pneumonia*	96.70	MH179986.1
*H. alvei*	96.70	Z83203.1
*R. planticola*	96.70	MW433720.1
*P. mirabilis*	96.51	LR738976.1
*C. freundii*	96.70	OP762964.1
*Enterobacter* spp.* ANT 02*	96.92	HM803168.1
*Enterobacter* spp.	96.70	MN208100.1
*E. hormaechei*	96.70	CP056649.1
*S. marcescens*	96.26	KJ179616.1
*P. stuartii*	96.04	LT899969.1
*P. rustigianii*	93.83	KJ638995.1

*E. hormaechei=Enterobacter hormaechei, E. cloacae=Enterobacter cloacae, S. plymutica=Serratia plymutica, P. vulgaris=Proteus vulgaris, M. morganii=Morganella morganii, K. pneumonia=Klebsiella pneumonia, H. alvei=Hafnia alvei, R. planticola=Raoultella planticola, P. mirabilis=Proteus mirabilis, C. freundii=Citrobacter freundii, S. marcescens=Serratia marcescens, P. stuartii=Providencia stuartii, P. rustigianii=Providencia rustigianii*

## Discussion

The initial histamine level in European pilchard stored under ice was negligible. At this temperature, there was no perceptible histamine production. However, histamine content increased rapidly during storage at 10°C and exceeded 2300 ppm at 22°C after 36 h of storage. These results are consistent with a study on the presence of histamine in canned sardines in Morocco, which showed that the histamine level was very low and did not exceed 5 ppm at refrigeration [14]. High levels of histamine were reported in a previous study at 30°C in Morocco [15]. Houicher *et al*. [15] reported a very high histamine induction power of spoiled sardine bacterial strains of about 5201 ppm for *P. mirabilis* and 2333 ppm for *E. cloacae*. These data are consistent with the results of rapid histamine evolution at 10°C and 22°C obtained in this study. Indeed, a rapid increase in histamine content at 10°C could be due to the effects of psychrophilic bacteria such as *Morganella psychrotolerans* and *Photobacterium phosphoreum*, which can develop at low temperatures (from 7°C) [19, 20]. In addition, the body temperature of sardines from temperate waters (Fishing area 13°C–20°C) can trigger histamine production quickly after capture by promoting and accelerating contamination of fish muscle. In this study, the multiplication rate of the APC population was 5.3 times between 48 and 96 h, 1.9 times between 96 and 144 h of storage at 10°C, 9.4 times between 12 and 24 h, and 2.1 times between 24 and 36 h of storage at 22°C, suggesting a positive correlation between APC and histamine content, as reported in several previous studies by Chong *et al*. [21], and Bita *et al*. [22].

For coliforms, the multiplication rate of the population was 27.2 times between 48 and 96 h and 9.4 times between 96 and 144 h of storage at 10°C. At 22°C, the multiplication rate was 51.3 times between 12 and 24 h and 2.7 times between 24 and 36 h of storage. These high multiplication rates explain the high levels of histamine in sardines stored at 10°C and 22°C, in addition to the histaminogenic power of isolated bacteria (*M. morganii, P. mirabilis* and *K. ozaenae* which are recognized as highly producing).

At 0°C, the absence of TTC belonging to Enterobacteriaceae, a microflora capable of producing histamine, explains the low histamine content, which did not exceed 5 ppm. These results can be explained by the proliferation of specific spoilage microorganisms responsible for organoleptic changes observed in sardines even at 0°C, especially in the intestinal region where bacterial degradation was more pronounced during spoilage, as confirmed by Akila and Sathi Kumaran [23]. The inhibition of histamine-producing bacteria follows competition with specific spoilage microflora. These findings were also confirmed by the results of the amplification of *hdc* genes that were negative for all strains isolated in sardine stored at 0°C, unlike storage at 10°C and at 22°C, where the level of histamine was exponential. The isolation rate of highly histamine-producing bacteria was high, and the results of amplification of *hdc* genes were positive (*M. morganii, P. mirabilis*, and *K. ozaenae*).

The results of bacterial identification and the identified bacteria indicate that non-*Enterobacteriaceae* bacteria may have grown in the VRBG medium. In fact, only 55/107 isolated bacteria were identified as *Enterobacteriaceae*. The genera *Citrobacter, Enterobacter, Shigella*, and *Serratia* were present at different storage temperatures and were studied with a variable prevalence rate. *E. coli* and *Proteus* were present during storage at 10°C and 22°C, whereas *Morganella* and *Klebsiella* were present only during storage at 22°C. The presence rate of the identified genera revealed differences in the distribution of bacterial genera according to temperature. These results show that temperature plays a crucial role in the composition of the bacterial population, influencing the presence and abundance of certain genera of bacteria. These results are consistent with other studies that confirmed the presence of a set of germs belonging to the *Enterobacteriaceae* family, particularly fecal contamination germs such as *E. coli* [24]. In addition, a positive correlation between Enterobacteria levels and histamine content was reported [25, 26].

The bacteria isolated in this study are histamine-producing species. *M. morganii* and *P. mirabilis* had the highest induction rates, whereas *K. ozaenae* and *S. plymuthica* had moderate induction rates. These results are generally consistent with those reported in previous studies [9–15], except for *Enterobacter*, which is considered a strong histamine producer [27], whereas in this study, its histamine induction power was very low.

However, different histamine production values have been reported in broth cultures. *E. cloacae* isolated from tuna produced 86.4 ppm, *P. mirabilis* isolated from mackerel produced 65.5 ppm, *S. liquefaciens* isolated from bonito produced 94 ppm, *and Shigella* produced 37.76 ppm [28]. Houicher *et al*. [15] reported a histamine induction power of about 5201 ppm for *P. mirabilis* and 2333 ppm for *E. cloacae*. Emborg *et al*. [29, 30] reported the histamine production of *M. psychrotolerans* in vacuum-packed cold-smoked tuna stored at 10°C over 6000 ppm and >3500 ppm at 5°C.

### Molecular biology

For phylogenetic analysis, universal primers specific to the *16S rRNA* gene were successfully amplified. Comparison and alignment of the sequences confirmed biochemical identification and membership of Enterobacteria families with high degrees of similarity (93.8%–96.98%). The *hdc* genes of the strains *M. Morganii*, *P. mirabilis*, and *K. ozaenae* were successfully amplified; the other strains did not generate amplification products. These results are consistent with those reported by Björnsdóttir-Butler *et al*. [31], who found that PCR fails to detect the presence of *hdc* gene in low histamine-producing bacteria. This was confirmed by the histamine content, which was too low at 0°C, and the nature of the bacteria isolated at this temperature, which are weakly producing histamine. Unlike *M. Morganii*, *P. mirabilis*, and *K. ozaenae*, the *hdc* gene was successfully detected and amplified and the levels of histamine production by these bacteria were important. The comparison of amplified *hdc* genes showed high similarity with those of *M. morganii*, with 98.67% for *M. morganii*, 98.52% for *P. mirabilis, and* 98.28% for *K. ozaenae*.

The blast of HDC genes in the NCBI database showed strong similarity with that of *M. morganii*, whose group of genes associated with HDC contains two histidine-bearing histamines located upstream and downstream of the *hdc* gene encoding amino-acid permease and followed by the *hisS* gene encoding a histidine tRNA ligase. A previous study by Ferrario *et al*. [32] has shown that *Klebsiella oxytoca* contains an identical organization of the *hdc* genes to that of *M. morganii* and that histamine formation could involve two enzymes: Histidine histamine, which brings histidine inside and excretes histamine outside, and HDC, which catalyzes decarboxylation.

## Conclusion

This study revealed a strong influence of temperature and storage duration on histamine content and microbial count. The microbiological profile during 0°C storage showed a moderate increase in APC, a decrease in coliforms, and the absence of TTC. At 10°C and at 22°C, notable proliferation of all microflora was observed. *P. rustigianii, M. morganii, K. ozaenae, S. plymuthica, C. Freundii, P. mirabilis, E. cloacae, and E. coli* were identified at varying rates of occurrence. Isolated strains exhibited different rates of histamine induction in a histidine broth. *M. Morganii* had the highest induction rate and could produce 1600 ppm *. P. mirabilis* could produce 256 ppm, *K. ozaenae* 136 ppm, and *S. plymuthica* 115 ppm.

The amplification of the *16S rRNA* gene and the alignment of the obtained sequences confirmed biochemical identification and the membership of the enterobacteria family with significant degrees of similarity (93.8%–96.98%). RT-PCR showed positive results for the presence of genes associated with HDC. The *hdc* genes of *M. morganii*, *P. mirabilis*, and *K. ozaenae* were successfully amplified and exhibited strong similarities with the reference strain of *M. morganii*.

Using molecular biology to identify bacterial species is an indispensable tool for exploring bacterial diversity. The results confirmed that most of our isolates belong to genera identified by biochemical methods, with significant degrees of similarity. Further studies should be conducted to sequence all genes involved in histamine production to understand their organization and role in histamine induction under different storage and processing conditions.

## Data Availability

The supplementary data can be available from the corresponding author upon a reasonable request.

## Authors’ Contributions

RK: Conceptualized and conducted the study, analyzed data, and wrote the manuscript. SD, OE, RA, and NB: Conceptualized the study, analyzed data, supervised the study, and revised the manuscript. All authors have read and approved the final manuscript.
